# Merkel Cell Carcinoma of the Left Cheek With Testicular Metastasis: An Uncommon Occurrence

**DOI:** 10.7759/cureus.42188

**Published:** 2023-07-20

**Authors:** Pokhraj P Suthar, Kranthi K Marathu, Jagadeesh S Singh, Avin Kounsal, Divya Saini, Lavanya Chhetri, Rameshwar Prasad, Mohamed Z Hussein, Gladson Scaria

**Affiliations:** 1 Department of Diagnostic Radiology and Nuclear Medicine, Rush University Medical Center, Chicago, USA; 2 Department of Public Health, Johns Hopkins Bloomberg School of Public Health, Baltimore, USA; 3 Department of Clinical Nutrition, Rush University, Chicago, USA; 4 Department of Pathology, Rush University Medical Center, Chicago, USA

**Keywords:** ultrasound (us), merkel cell carcinoma, pathology, 18f-fdg pet/ct scan, ct

## Abstract

Merkel cell carcinoma (MCC) is an infrequent and aggressive neuroendocrine tumor of the skin. 18F-fluorodeoxyglucose positron emission tomography/computed tomography (18F-FDG PET/CT) is an effective imaging technique with good diagnostic accuracy that may be used to help stage MCC and for detecting unexpected recurrences and distant metastatic disease. Other causes of testicular neoplasms, such as primary testicular tumors, lymphomas, or anaplastic small cell melanomas, are difficult to differentiate from MCC testicular metastases on imaging, and tumor markers and histopathology will help confirm it. The current case is a 65-year-old non-immunocompromised male with Merkel cell carcinoma who was incidentally identified with testicular metastases on PET/CT and confirmed on histopathology.

## Introduction

Merkel cell carcinoma (MCC) has a high risk of local recurrence and distant metastasis, resulting in a poor prognosis [1-3]. Recent advances in immunotherapy have improved the survival of patients with metastatic MCC, notably with the use of anti-programmed death ligand 1 (PDL1) as a first-line treatment. Although MCC may spread to numerous anatomical places, it has more unique and aggressive characteristics than other cancers. Testicular metastasis is extremely uncommon, accounting for just a small fraction of cases documented in the literature [4]. There have only been 11 recorded cases of MCC metastasizing to the testis in the literature [5-14]. In this report, we present the 12th case and provide a comprehensive review of the existing literature.

## Case presentation

A 65-year-old non-immunocompromised male was sent to Rush University Medical Center for biopsy-proven Merkel cell carcinoma of the left cheek. For four months, the patient experienced a growing left cheek bulge. The patient denied any history of hoarseness, dysphagia, shortness of breath, or fever. He had a history of essential hypertension and diabetes mellitus and was taking antihypertensive and hypoglycemic medications orally. Except for borderline hypertension (blood pressure: 130/84 mmHg), his vital signs were normal (temperature: 97.6°F (36.4°C), heart rate: 74 beats per minute, respiration rate: 17 beats per minute, and SpO_2_: 99%). On physical examination, a 5 cm subcutaneous lump was visible in the left malar eminence abutting the left lower eyelid, with recent biopsy alterations.

**Figure 1:**
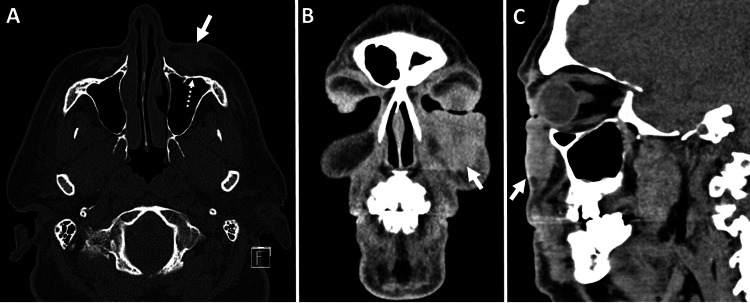
Unenhanced CT maxillofacial (A) axial image in bone algorithm and (B) coronal and (C) sagittal images in soft tissue algorithm demonstrating a 3.2 × 1.8 × 3.2 (T, AP, CC) cm soft tissue lesion in the skin and subcutaneous plane anterior to the left maxillary sinus (white arrow in A, B, and C). There is no evidence of underlying maxillary sinus erosion or bony invasion (dashed white arrow in A). CT: computed tomography, T: transverse, AP: anteroposterior, CC: craniocaudal

**Figure 2:**
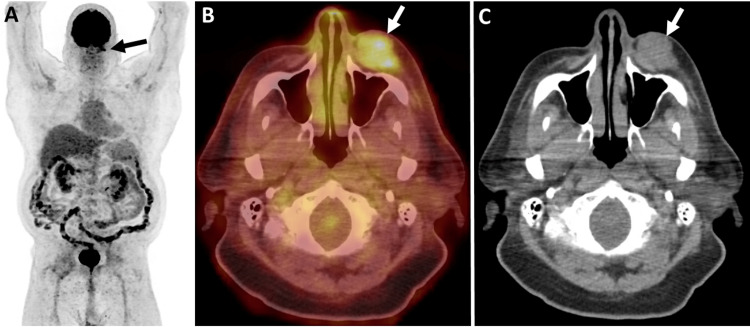
Initial staging 18F-FDG PET/CT: (A) maximum intensity projection, (B) trans-axial fused PET/CT, and (C) unenhanced axial CT images demonstrating focal increase FDG uptake in the left face (black arrow in A) corresponding to the metabolically active skin and subcutaneous soft tissue lesion anterior to the left maxillary sinus without invasion in the left maxillary sinus (white arrow in B and C). There is no other abnormal metabolic active lesion in the whole body (A). 18F-FDG PET/CT: 18F-fluorodeoxyglucose positron emission tomography/computed tomography

**Figure 3:**
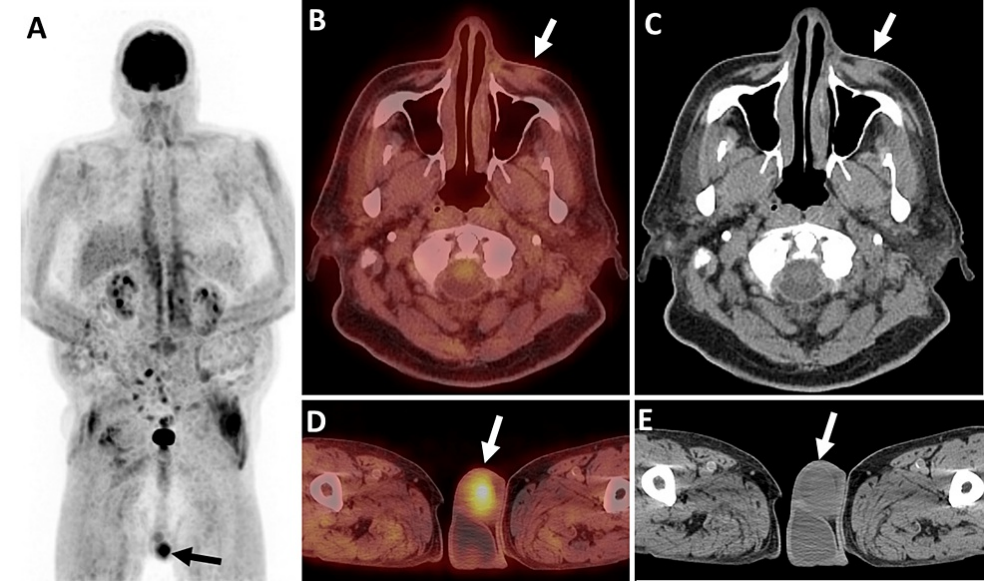
Re-staging 18F-FDG PET/CT: (A) maximum intensity projection, (B and D) trans-axial fused PET/CT, and (C and E) unenhanced axial CT images demonstrating significant interval decrease in size and FDG uptake of the left premaxillary soft tissue (SUVmax 1.5, white arrow in B and C) without hypermetabolic lymph nodes in the neck, chest, and abdomen. Incidentally seen is an asymmetrical intense FDG uptake in the left testicle (SUVmax 10.3, white arrow in D and E). 18F-FDG PET/CT: 18F-fluorodeoxyglucose positron emission tomography/computed tomography

**Figure 4:**
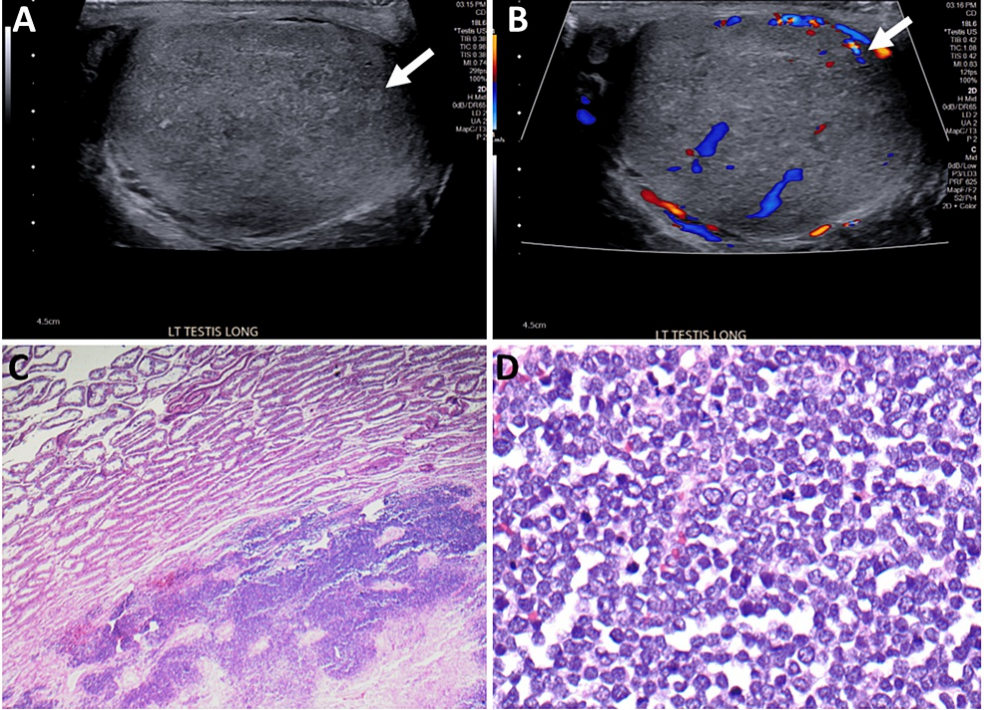
(A and B) Ultrasound color duplex image of the left scrotum demonstrating a heterogenous appearance (white arrow in A) with increased vascularity of the left testis (white arrow in B) concerning testicular neoplasm. (C and D) Photomicrograph of the left testis after left orchidectomy (hematoxylin and eosin stain: ×2 (C) and ×40 (D)). The cells were small, round, and blue with stippled chromatin and inconspicuous nucleoli.

An initial computed tomography (CT) maxillofacial scan was performed to examine the lesion and underlying locoregional anatomy. On CT, there was a 3.2 × 1.8 × 3.2 (transverse (T), anteroposterior (AP), craniocaudal (CC)) cm soft tissue lesion in the skin and subcutaneous plane anterior to the left maxillary sinus with no erosion or invasion of the underlying bone (Figure 1). A staging 18F positron emission tomography/computed tomography (PET/CT) revealed a soft tissue mass in the left malar eminence with fluorodeoxyglucose (FDG) uptake (SUV_max_ 7.2) with no nodal or distant metastases (Figure 2). The patient received 60 Gy of radiation treatment in 30 fractions. A four-month follow-up CT of the neck soft tissue revealed a reduction in the nodular, ill-defined, infiltrative left cheek soft tissue mass lesion, indicating a favorable local response. Restaging 18F-FDG PET/CT indicated a substantial interval reduction in size and FDG uptake of left premaxillary soft tissue (SUV_max_ 1.5) without hypermetabolic lymph nodes in the neck, chest, and abdomen. Incidentally, an asymmetrical intense FDG uptake was seen in the left testicle (SUV_max_ 10.3) (for reference, the SUV_max_ of the right testicle was 2.9) (Figure 3). An ultrasound (US) of the scrotum was performed to characterize the testicular lesion. The US revealed an enlarged left testicle with a heterogenous appearance and increased vascularity concerning the testicular neoplasm (Figure 4A, 4B). The patient underwent a left orchiectomy. Pathological examination showed a tan-red, soft, ill-defined mass in the left testicle, occupying approximately 90% of the left testicle. Histopathological analysis revealed a metastatic Merkel cell tumor infiltrating the left testis. The cells were tiny, spherical, and blue, with stippled chromatin and inconspicuous nucleoli (Figure 4C, 4D). The lesion showed that cytokeratin (CK) 20, synaptophysin, and chromogranin were positive in tumor cells, whereas cluster of differentiation (CD) 99, CD3, CD20, alpha-fetoprotein, placental alkaline phosphatase (PLAP), and beta-human chorionic gonadotropin (hCG) were negative, validating the diagnosis of metastatic Merkel cell carcinoma. The patient received pembrolizumab for metastatic disease and palliative abdominal and pelvic radiotherapy for metastases in the right proximal thigh and retroperitoneum. Following that, the patient survived for another two months.

## Discussion

Merkel cell carcinoma is a rare and aggressive neuroendocrine neoplasm of the skin that commonly affects the head and neck region [1]. MCC predominantly affects older individuals, particularly males, and excessive exposure to ultraviolet light and immune suppression are considered significant risk factors [9,15]. The exact pathogenesis of MCC remains uncertain, although it has been associated with polyomavirus.

MCC is most found in the skin of the head and neck, followed by the upper limbs and shoulders. MCC has been on the rise in recent years, especially among white people with sunburned skin [1]. At the time of diagnosis, around 30% of patients already have occult metastatic spread, predominantly in lymph nodes. The liver, bone, lung, brain, adrenal gland, and skin are common sites of distant metastasis, although metastasis to the genitourinary system is uncommon [2]. Merkel cell carcinoma metastasis to the testes is an extremely rare event, with just 11 cases reported in the medical literature [5-14]. On imaging, it is difficult to differentiate MCC metastasis from other testicular neoplasms such as primary testicular tumors, synchronous undetected small cell lung cancer, lymphomas, or anaplastic small cell melanomas. Tumor markers are useful in differentiating these disorders. The presence of neuroendocrine markers (synaptophysin, chromogranin, and CD56) as well as the unique dot-like pattern of CK20 immunoexpression (without CK7 immunoexpression) helps confirm the diagnosis. Small lung cell carcinoma metastasis, on the other hand, expresses CK7, lymphoma expresses CD45, and melanoma metastases express S100 and HMB-45 Melan-A [10].

18F-FDG PET/CT scan is a very useful imaging technique in whole-body staging of MCC. It is also useful for detecting unexpected recurrences and distant metastatic disease.

MCC is an aggressive tumor that responds favorably to chemotherapy and radiation treatment. Relapses are prevalent, as are regional and distant metastases [12]. The patient in this case was diagnosed with a localized stage of cancer with no nodal or distant metastases and was treated with radiation. The patient responded well to radiotherapy locally, but metastases to the bone and testis occurred. The blood-testis barrier, Sertoli cells, and immunoregulatory systems all have an important role in the prevention of testicular involvement.

A review of the current literature on Merkel cell cancer with testicular metastasis is depicted in Table 1. The current case is compared to the previously reported cases in the literature. Most of the previously reported cases had initial tumors involving the extremities, except for one case involving the upper lip, and none involving the cheek. In this case, the time to metastasis is similar to the example described by Lara Moscoloni et al. [6]. Two more cases described by Ro et al. [14] and Tummala et al. [10] began with localized lesions and advanced to distant involvement when testicular metastasis was discovered. However, none of them developed simultaneous bone metastases as evident in our case. The cases described by Ro et al. [14] and Tummala et al. [10] received medical therapy after orchidectomy, and their survival durations were six and 14 months, respectively. In the current case, the survival rate was only two months. Each patient received specific palliative therapy. Four cases with a single testicular metastasis and no additional extra-testicular tumors survived major surgery for 12 months with no subsequent relapses.

**Table 1:** Reported cases of Merkel cell carcinoma with testicular metastasis in the literature: clinical characteristics, staging, treatment, and prognostic features. FDG: fluorodeoxyglucose, RT: radiotherapy, CT: chemotherapy, N/A: not available, LN: lymph node, M1: metastatic stage, PR: primary tumor resection, MCC: Merkel cell carcinoma, SRS: somatostatin receptor scintigraphy

Author	Clinical features	Age	Side	Primary MCC	Rx for primary	Rx testicular lesion	Staging at diagnosis	Staging during the time of testis metastasis detection	Time to testis metastasis	Order of events
Present case	Incidental FDG uptake in the testis	65	Left	Left cheek	RT	Orchiectomy	Local	None	4 months	RT - orchiectomy - immunotherapy
Laffi et al. (2022) [5]	Testicular mass	57	Right	Left knee	Resection + adjuvant RT	Immunotherapy	Local	Bone, subcutaneous, and peritoneal - M1	51 months after primary	PR - orchiectomy -immunotherapy
Lara Moscoloni et al. (2020) [6]	Painful testicular swelling	65	Right	Right thigh	Surgery CT + RT	Orchiectomy + immunotherapy	Local	None	1 month	PR - CT - RT
Gigliano et al. (2020) [7]	Scrotal edema	58	Left	Left wrist	Local excision	Radical orchiectomy	Locally advanced	LN	37 months after primary	LR - orchiectomy
Mweempwa et al. (2016) [8]	Painless testicular swelling	66	Right	Unknown	N/A	Orchiectomy + CT	Advanced	None	N/A	Orchiectomy - CT
Whitman et al. (2007) [9]	Testicular mass	70	Right	Right gluteus	Resection + LN dissection	Orchiectomy	Locally advanced	None	15 months	PR - orchiectomy
Tummala et al. (2006) [10]	Testicular mass	54	Right	Left forearm	Resection + RT + CT	Orchiectomy	Local	None	2 months	PR - orchiectomy
Schwindl et al. (2006) [11]	Painful testicular swelling	70	Right	Right forearm	Resection + RT	Orchiectomy	Locally advanced	LN - M1	7 months	PR - orchiectomy -CT
Gleason et al. (2006) [12]	Painless Testicular mass	53	Left	Right gluteus	CT + RT	Orchiectomy	Advanced	Shoulder, lung, mediastinal LN - M1	22 months	PR - metastasis - RT - orchiectomy -metastasis - RT
Rufini et al. (2004) [13]	Incidental SRS uptake in the testis	38	Bilateral	Unknown	N/A, received CT	None	Advanced	Abdominal LN - M1	2 months	Metastasis biopsy - CT
Ro et al. (1990) [14]	Painless enlargement	73	Right	Upper lip	Resection + RT + iridium implant	Orchiectomy + CT	Locally advanced	LN, liver, subcutaneous - M1	18 months	PR - metastasis
Ro et al. (1990) [14]	Testicular swelling	47	Bilateral	Left elbow	Resection + RT + CT	Bilateral orchiectomy	Local	None	12 months	PR - orchiectomy

## Conclusions

In conclusion, Merkel cell carcinoma (MCC) is an infrequent and aggressive neuroendocrine tumor of the skin and can manifest with distant metastasis. Merkel cell carcinoma metastasis to the genitourinary tract and particularly involvement of the testis is very rare. Clinicians should be aware of the potential testicular involvement in MCC to direct appropriate investigation and timely management. 18F-FDG PET/CT plays an important role in whole-body staging and detecting local and distant metastasis in MCC and may be helpful for detecting incidental testicular involvement. Multidisciplinary management is beneficial in cases of advanced-stage disease, and immunotherapy appears to hold promise.
